# A review on knowledge and information extraction from PDF documents and storage approaches

**DOI:** 10.3389/frai.2025.1466092

**Published:** 2025-09-05

**Authors:** Salvador D. Atagong, Henri Tonnang, Kennedy Senagi, Mark Wamalwa, Komi M. Agboka, John Odindi

**Affiliations:** ^1^Data Management and Geo-Information Unit (DMMGU), International Centre of Insect Physiology and Ecology, Nairobi, Kenya; ^2^Department of Environmental Science, University of KwaZulu Natal, Durban, South Africa; ^3^International Institute of Tropical Agriculture (IITA), Ibadan, Nigeria

**Keywords:** natural language processing, large language models, knowledge base, knowledge extraction, knowledge graphs

## Abstract

**Introduction:**

Automating the extraction of information from Portable Document Format (PDF) documents represents a major advancement in information extraction, with applications in various domains such as healthcare, law, or biochemistry. However, existing solutions face challenges related to accuracy, domain adaptability, and implementation complexity.

**Methods:**

A systematic review of the literature was conducted using the Preferred Reporting Items for Systematic Reviews and Meta-Analyses (PRISMA) methodology to examine approaches and trends in PDF information extraction and storage approaches.

**Results:**

The review revealed three dominant methodological categories: rule-based systems, statistical learning models, and neural network-based approaches. Key limitations include the rigidity of rule-based methods, the lack of annotated domain-specific datasets for learning-based approaches, and issues such as hallucinations in large language models.

**Discussion:**

To overcome these limitations, a conceptual framework is proposed comprising nine core components: project manager, document manager, document pre-processor, ontology manager, information extractor, annotation engine, question-answering tool, knowledge visualizer, and data exporter. This framework aims to improve the accuracy, adaptability, and usability of PDF information extraction systems.

## 1 Introduction

Natural language has been employed for centuries to convey information and knowledge, primarily through printed documents such as the Bible, the Koran, and several mythologies and civilization archives. For many years, conserving these physical documents has been challenging due to inherent vulnerabilities that include sensitivity to temperature, paper degradation, and fires. However, in the recent past, digital documents have become increasingly popular due to their space-saving, ease of sharing, and enhanced security features. According to Johnson (2021), the Portable Document Format (PDF) is one of the most widely used formats for digital documents, accounting for more than 83% of documents shared over the web (Johnson, 2021). In comparison to physical documents, this prevalence can be attributed to their platform independence and the ability to preserve original document formats. According to Abdillah (2013); Nganji (2015); Axel Newe (2018), PDFs account for a significant portion of scholarly documents, while (Bornmann and Mutz, 2015) notes that their creation rate has grown exponentially over the years. This growth has meant that the task of collecting and extracting specific information from a large volume of PDF documents has become arduous and time-consuming.

Many studies (Gupta et al., 2022; Abdollahi et al., 2021; Guan et al., 2022; Nundloll et al., 2022) have endeavored to address the challenge of automatically extracting specific information from PDF documents. These efforts primarily leverage Natural Language Processing (NLP) algorithms and Optical Character Recognition (OCR) techniques. NLP encompasses a set of computational techniques designed for the automatic analysis and representation of human languages grounded in theoretical foundations as emphasized by Chowdhary (2020). These techniques have been extensively adopted to extract information from a wide range of written sources, facilitating the discovery of new and previously undisclosed information in textual data (Chen et al., 2022). According to Chowdhary (2020); Abdullah et al. (2023), Information Extraction (IE) in literature has been broken into many sub-tasks namely; (1) Named Entity Recognition (NER) that aims to extract named entities from a given text corpus; (2) Relationship Extraction (RE) that focuses on extracting relationships between the named entity of a given corpus; (3) Question Answering (QA) aimed at answering natural language questions and highly dependent on the two previous sub-tasks; (4) Knowledge Extraction dedicated to building a knowledge base from a text corpus; (5) Event Extraction (EE) aimed at identifying events and all their properties (e.g. organizer, time) from a text corpus; and (6) Causality Extraction (CE), that aims to extract cause-effect relations between pairs of labeled nouns from text (Yang et al., 2022). Furthermore, IE approaches have been generally classified into three categories, namely, rule-based approaches, statistical learning-based approaches, and neural network approaches Abdullah et al., 2023; Mannai et al., 2018). To gain a comprehensive understanding of how IE is performed from PDFs, specifically, how people go from PDF documents to structured databases and identify the challenges encountered along with potential unaddressed gaps, we structured our review around the following research questions:

1. What motivates information or knowledge extraction from PDF documents?
2. Which techniques or algorithms are used for automated information extraction, and what difficulties are encountered?
3. How is this extracted information structured for further processing and analysis?
4. How are the performance and effectiveness of these techniques or algorithms evaluated?
5. In what ways is the extracted information or knowledge stored and represented?

To provide an answer to these questions, our study further provides a comprehensive overview of recent developments in the field of IE from PDF documents, focusing on research published between 2017 and 2025. During this timeframe, significant advancements have been made in the field of NLP, with the introduction of Transformers (Vaswani et al., 2017) and Language Models (Huguet Cabot and Navigli, 2021; Devlin et al., 2019). This study aims to not only delineate the current trends in these information extraction techniques but also to identify persisting challenges. Furthermore, we propose a conceptual framework for automatic information extraction from text and structuring, which amalgamates language models with common ontologies to fine-tune and oversee the entire extraction process, to enhance its adaptability across diverse domains.

The subsequent sections of the manuscript are organized as follows: Section Section 2 acknowledges previous work and contextualizes our review, Section 3 presents the methodology we used to select resources from published literature, Section 4 provides a summary and classification of the selected studies in the literature, in Section 5 we discuss our findings, Section 6 introduces an innovative conceptual framework for information and knowledge extraction, while Section 7 concludes the review.

## 2 Previous works

The field of Information Extraction (IE) continues to be highly active, with numerous reviews offering insights into its development over time. In this context, we highlight two particularly relevant studies.

Abdullah et al. (2023) presents a comprehensive review of IE applied to textual data, covering methodologies, applications, trends, and challenges from 2017 to 2022. The review underscores the critical role of IE in handling diverse textual sources and notes the growing reliance on deep learning methods to overcome the limitations of traditional and classical machine learning techniques. It introduces key concepts, recent innovations, and real-world applications, particularly in domains such as business and investment; while identifying persistent challenges such as data inconsistency, model selection difficulties, and algorithmic errors. The study offers practical guidance for researchers and outlines future directions, including the development of accessible evaluation tools and the exploration of under-researched application areas beyond the medical and biomedical domains.

In a more domain-specific context, Kononova et al. (2021) explores the use of text mining (TM) and natural language processing (NLP) in materials science. The review highlights the challenges of extracting structured insights from unstructured scientific literature, emphasizing that standard NLP tool, typically trained on general language data struggle with the specialized vocabulary of scientific publications. The authors survey recent advancements in TM and NLP tailored to materials science, discuss widely adopted techniques and notable case studies, and identify key technical hurdles, such as converting diverse document formats into raw text, achieving accurate sentence segmentation and parsing, and developing effective named entity recognition systems for chemical and material entities. This review is aimed at researchers seeking to understand the application of TM within scientific literature.

Building on these prior efforts and aiming to provide new insights rather than reiterating existing findings, our review focuses specifically on the processing of PDF documents for IE. Unlike the domain-specific perspective of Kononova et al. (2021) or the broader text-centric view of Abdullah et al. (2023), our approach is domain-independent. In addition, we place particular emphasis on the structuring and storage of extracted information, an area that, to the best of our knowledge, has not been thoroughly examined in existing literature.

## 3 Research methodology

We adopted the Preferred Reporting Items for Systematic Reviews and Meta-Analyses (PRISMA) (Liberati et al., 2009) methodology to derive and analyze existing literature on IE from PDF documents. The PRISMA methodology delineates itself in four main steps, namely: identification, screening, eligibility, and inclusion.

### 3.1 Identification

The identification step focused on identifying relevant studies that discuss the extraction of information from PDF documents. A search was conducted from January 2017 to May 2025 using the advanced search function of selected scholar databases, namely Web of Science, IEEE Explore, and PubMed. Our search strategy included a combination of keywords listed in Table 1, categorized in text processing-related keywords and document type-related keywords. Considering the syntactic specificity of each scholar's databases, different search queries were elaborated to perform the search as shown in Table 2.

**Table 1:** Keywords selected for retrieving relevant studies from online scholar databases.

**Text processing**	**Document type**
Information extraction, knowledge extraction, nlp, natural language processing, named entity recognition, named entity extraction, relation extraction, relationship extraction, event extraction,	Unstructured document, portable document format, pdf

**Table 2:** Search queries utilized on each of the selected online scholar databases.

**Database**	**Search query**
Web of Science	((TI=(information extraction *) OR AB=(information extraction *)) OR (TI=(knowledge extraction *) OR AB=(knowledge extraction *)) OR (TI=(NLP *) OR AB=(NLP *)) OR (TI=(natural language processing *) OR AB=(natural language processing *)) OR (TI=(named entity recognition *) OR AB=(named entity recognition *)) OR (TI=(named entity extraction *) OR AB=(named entity extraction *)) OR (TI=(relation extraction *) OR AB=(relation extraction *)) OR (TI=(event extraction *) OR AB=(event extraction *))) AND ((TI=(unstructured document) OR AB=(unstructured document)) OR (TI=(portable document format) OR AB=(portable document format)) OR (TI=(PDF document) OR AB=(PDF document))) AND (DOP=(2017-01-01/2025-05-30))
IEEE Xplore	((“Publication Title”:information extraction) OR (“Abstract”:information extraction) OR (“Publication Title”:knowledge extraction) OR (“Abstract”:knowledge extraction) OR (“Publication Title”:NLP) OR (“Abstract”:NLP) OR (“Publication Title”:natural language processing) OR (“Abstract”:natural language processing) OR (“Publication Title”:named entity recognition) OR (“Abstract”:named entity recognition) OR (“Publication Title”:named entity extraction) OR (“Abstract”:named entity extraction) OR (“Publication Title”:relation extraction) OR (“Abstract”:relation extraction) OR (“Publication Title”:relationship extraction) OR (“Abstract”:relationship extraction) OR (“Publication Title”:event extraction) OR (“Abstract”:event extraction)) AND ((“Publication Title”:Unstructured document) OR (“Abstract”:Unstructured document) OR (“Publication Title”:portable document format) OR (“Abstract”:portable document format) OR (“Publication Title”:PDF document) OR (“Abstract”:PDF document))
PubMed	((Information extraction[Title/Abstract]) OR (knowledge extraction[Title/Abstract]) OR (NLP[Title/Abstract]) OR (natural language processing[Title/Abstract]) OR (named entity recognition[Title/Abstract]) OR (named entity extraction[Title/Abstract]) OR (relation extraction[Title/Abstract]) OR (relationship extraction[Title/Abstract]) OR (event extraction[Title/Abstract])) AND ((Unstructured document[Title/Abstract]) OR (portable document format[Title/Abstract]) OR (PDF document[Title/Abstract])) AND ((“2017/01/01”[Date - Publication] : “2025/05/30”[Date - Publication]))

### 3.2 Screening

In order to drill down the initial pool of publications to ensure the quality and relevance of the selected publications, the first screening process was performed on the titles of the collected items. At this stage, reviews and articles whose titles did not meet our focused scope were removed. The second screening phase used the inclusion and exclusion criteria listed in Table 3 to further refine the selection. We reviewed the titles and abstracts of the kept studies, focusing on aspects such as clarity of the methodology and alignment of the study objectives with our research objectives. The results were stored in a Microsoft Excel sheet to keep track of filtered articles, perform basic operations such as duplicate deletion, and perform quick analysis of the selected studies.

**Table 3:** Inclusion and exclusion criteria utilized for refining the selection of studies relevant to our review.

**Inclusion**	**Exclusion**
• Publication year between 2017 and 2025 • Publication is in the English language • Article is open access • Study focuses on information extraction and structuring from PDF documents • Studies that elaborate on how the extracted data is stored	• PDF doesn't mean Portable Document Format (e.g., Probability Density Function) • Article is a review

### 3.3 Eligibility and quality assessment

To further refine our selection, we adopted a rating methodology derived from the work of Abdullah et al. (2023) to formalize the selection process by quantifying the quality of examined papers. As shown in Table 4, we established a structured questionnaire to evaluate each publication and retained only papers with a score greater than 4, with a minimum score of 1 on the first question and at least 0.5 on the fourth question (see Equation 1). The rationale for these values is rooted in the criteria detailed in Table 4. Specifically, a score of 1 on the first question indicated that the study's objectives were clear and focused on IE, while a 0.5 score on the fourth question indicated that the study had at least proposed an evaluation of its performance. In addition, a total of at least 4 ensured that the IE methodology was clearly outlined within the study.


(1) $$\forall i\in \{1,2,3,4,5\};\forall x\in P;P_{k}\cup \{x\}\Leftrightarrow (\sum_{i=1}^{5}\geq 4);\{\begin{array}{l} S_{1}=1\\ S_{4}\geq 0.5 \end{array}$$

Where: *S*_*i*_ stands for a score on the *i*^*th*^ question (*C*_*i*_ from Table 4)

P is the set of initial papers

$P_{k}$ is the set of kept papers

**Table 4:** Articles rating scale against research objectives.

**Code**	**Criteria**	**Score**	**Description**
C1	Does the study define clear objectives, and do they meet our research question?	1	Yes, the study presents clear objectives and goals, which are clearly related to information extraction from text.
		0.5	The study presents its objectives, but the end goal is not information extraction, even though it somehow intervenes.
		0	The study does not clearly define its objectives.
C2	Does the study present a clear methodology?	1	Yes, the methodology of information extraction is clearly defined.
		0.5	The methodology is superficial or incomplete.
		0	No, the study does not present its methodology.
C3	Does the study present limitations?	1	Yes, the study presents its limitations in detail.
		0.5	The study states its limitations but not in detail.
		0	No, the study does not state its limitations.
C4	Does the study evaluate its performance?	1	Yes, the study is evaluated clearly using common metrics and compared to state-of-the-art methodology results in the field.
		0.5	The study is evaluated but no clear metrics are provided nor clear comparison with other methodologies.
		0	No metric is provided for study evaluation.
C5	Does the study handle the storage aspect?	1	Yes, the study presents the storage approach used to structure the saved information in detail.
		0.5	The study superficially talks about the restructuring of the extracted information, but no further details are provided.
		0	The study does not talk about how the extracted information is stored.

## 4 Results

**Figure 1:**
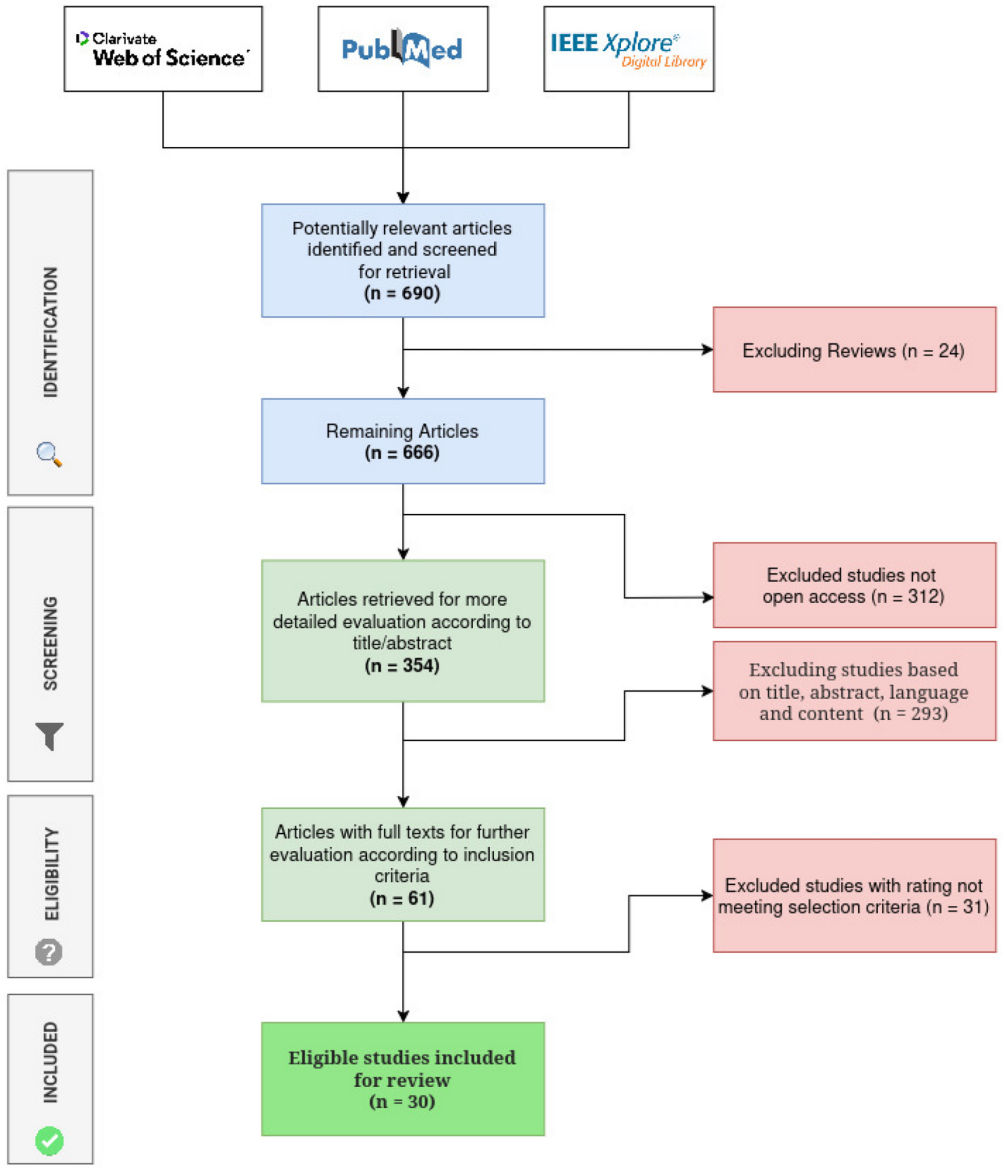
A summary of the PRISMA steps to find the articles that were reviewed.

**Figure 2:**
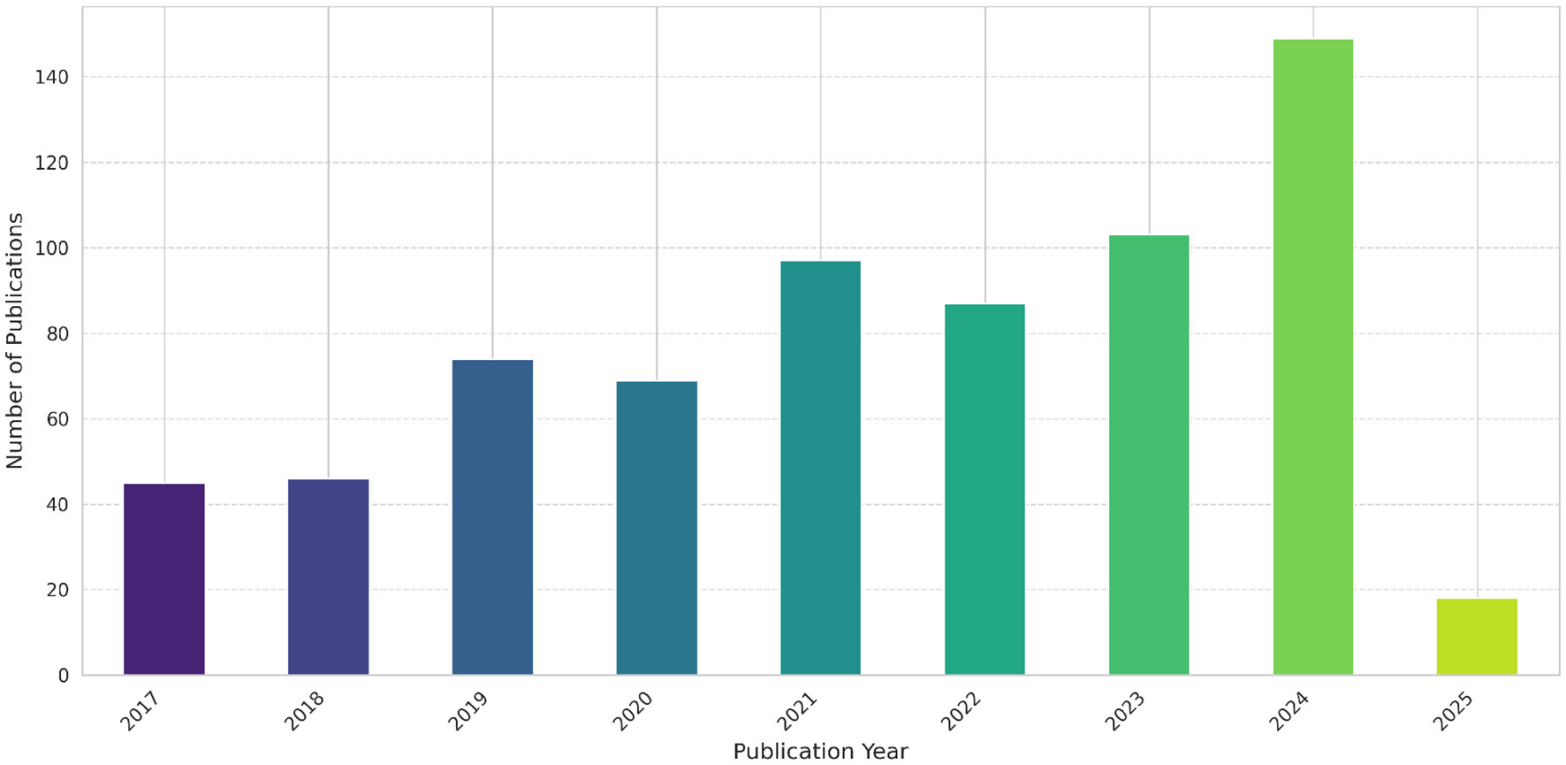
Initial distribution of fetched publications over the years.

**Figure 3:**
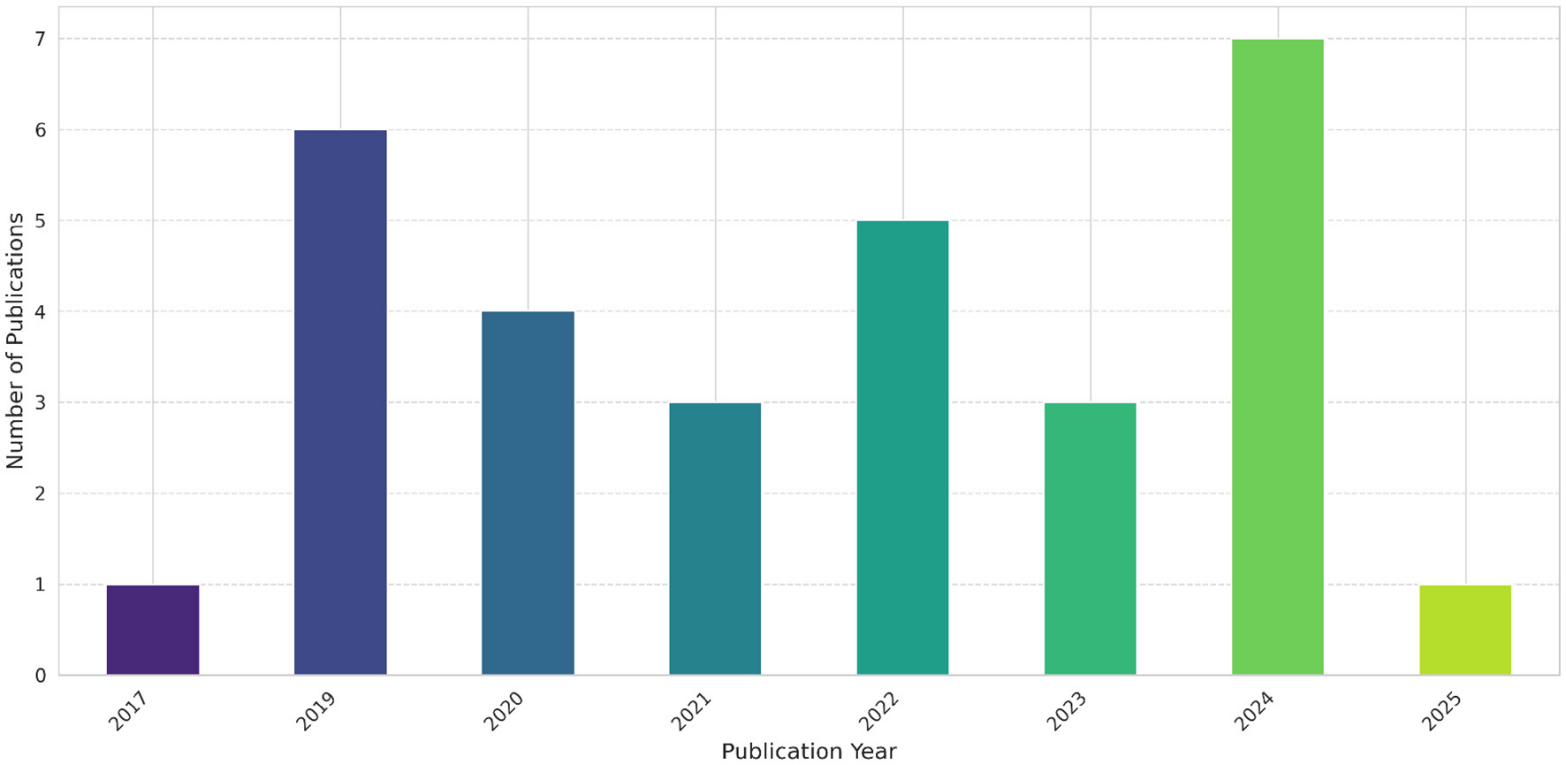
Distribution of selected publications over the years.

A comprehensive search of existing literature using the PRISMA methodology resulted in 690 unique articles from the selected scholarly databases (Web of Science, PubMed, and IEEE Explore). The filtering process was summarized in Figure 1, illustrating the evolution of the articles dataset from initial to final selection, after application of different screening, inclusion (see Figure 2), and exclusion criteria, followed by an eligibility assessment step. The screening process yielded 63 articles, while the eligibility assessment phase resulted in a final set of 30 articles (see Figure 3), which were deemed eligible for more in-depth examination (see Appendix Table A1).

## 5 Discussion

The analysis of the included studies revealed a vibrant and evolving landscape in the field of automated information extraction from PDF documents. Researchers are employing a diverse array of techniques, often combining approaches to tackle the inherent complexities of different document layouts and domains. However, despite significant advancements, the field continues to grapple with persistent limitations and challenges.

### 5.1 Motivations of information extraction from PDF documents

This study sought to explore the motivations for the automatic extraction of information from PDF documents and to track its evolution from 2017 to 2025. Our findings revealed significant emphasis on IE across various domains over the study period considered. It comes out that IE from PDF documents can be motivated by four key reasons: (1) Time optimization in critical tasks, such as medical records analytics (Chen et al., 2022; Jantscher et al., 2023; Meystre et al., 2017), (2) specific IE from large documents volumes (Papadopoulos et al., 2020; Dong et al., 2021) or automatic report analysis (Cho et al., 2023; Khandokar and Deshpande, 2024), (3) Knowledge discovery to help decision making (Adamson et al., 2023; Jaberi-Douraki et al., 2021; Xu et al., 2024), and (4) Building a structured databases for targeted information retrieval and analytics (Nundloll et al., 2022; Parrolivelli and Stanchev, 2023; Salamanca et al., 2024). We observed a shift in methodologies over time, with early studies favoring rules-based approaches and recent studies increasing adoption of automatic training approaches, capitalizing on pre-trained Language Models (LMs) and Large Language Models (LLMs) based on the transformer's architecture (Vaswani et al., 2017). This gradual shift can be attributed to the ease of adoption and adaptability (Huguet Cabot and Navigli, 2021; Kalyan, 2024; Bai et al., 2022) offered by automatic learning approaches compared to rule-based methods, which are expert-dependent, domain-specific, and less flexible (Turner et al., 2022).

### 5.2 Methods and approaches used to realize IE from PDF documents

In recent years, there has been a clear evolution in the methodologies used for information extraction (IE), particularly in text-rich domains such as healthcare, scientific literature, and administrative documentation. Initially, from around 2017 to 2019, rule-based approaches dominated the landscape. These systems relied heavily on handcrafted rules, domain-specific gazetteers, dictionaries, and ontologies to extract structured data from unstructured text. While effective within narrowly defined scopes, such systems required significant manual curation and were labor-intensive to adapt to new domains or languages. However, the field has undergone a significant transformation with the advent of neural network-based natural language processing (NLP), particularly with the introduction of pre-trained language models such as BERT (Siciliani et al., 2024; Tao et al., 2024), SciBERT (Gemelli et al., 2022), and GPT-4 (OpenAI et al., 2024). These models, trained on vast corpora, are capable of capturing nuanced linguistic patterns and semantic relationships, making them highly adaptable across various tasks and domains. Their generalizability and performance have led to a shift away from traditional rule-based or statistical methods toward more automated and scalable solutions.

Core NLP tasks, including tokenization, part-of-speech tagging, named entity recognition (NER), and relation extraction, remain foundational across applications and are routinely employed in pipeline architectures to structure information from unstructured text. This is evident in studies such as Becker et al. (2019), where such techniques are used to annotate clinical data with UMLS concepts, as well as in other works focusing on disease surveillance, patient information extraction, and biomedical literature analysis (Abulaish et al., 2019; Siciliani et al., 2024; Palm et al., 2019). The trend toward more sophisticated NLP models is further reflected in the application of deep learning architectures such as convolutional neural networks (CNNs) and long short-term memory networks (LSTMs), enabling tasks ranging from entity recognition in multimodal contexts to end-to-end extraction without the need for extensive manual annotation (Cho et al., 2023; Cesista et al., 2024). In parallel, semi-supervised approaches like self-training are being explored to address the scarcity of labeled data (Cesista et al., 2024; Salamanca et al., 2024; Tian et al., 2024).

For documents where visual structure plays a critical role, such as scanned forms, financial statements, or documents with complex layouts, computer vision techniques, including Optical Character Recognition (OCR), layout analysis, have become essential components of the IE pipeline (Khandokar and Deshpande, 2024; Li et al., 2019). Furthermore, the integration of domain ontologies and knowledge graphs continues to offer valuable structure and interpretability, guiding the extraction process and enabling downstream applications like knowledge base population (Yehia et al., 2019; Scannapieco and Tomazzoli, 2024). To support these diverse technical components, researchers increasingly employ modular, pipeline-based system architectures that allow for flexible integration of NLP, computer vision, and domain knowledge resources. These architectures are often underpinned by the creation of large, well-annotated datasets, which are recognized as essential for training, validating, and benchmarking the performance of modern, machine-learning-driven information extraction systems.

**Figure 4:**
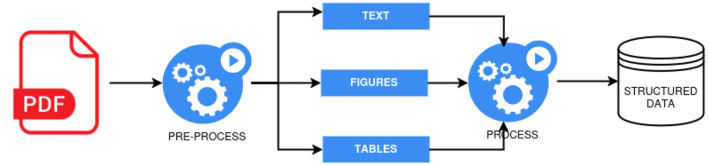
Illustration of the generic approach to IE from PDF documents, derived from examined studies.

As illustrated in Figure 4, the process of extracting information from PDF documents typically involves three key stages: pre-processing, processing, and storage. The pre-processing stage focuses on extracting and preparing the content of the PDF, targeting specific elements such as textual content, figures, tables, or combinations thereof for subsequent analysis. Due to the inherent challenges of directly processing PDF documents, various techniques have been developed and discussed in the literature. One such technique is Optical Character Recognition (OCR), a neural network-based approach, with Tesseract being a widely used library. Additionally, several PDF-specific libraries, such as PyMuPDF (Tao et al., 2024), PDFX (Ahmed and Afzal, 2020), XPDF (Li et al., 2019), and PDFMiner, are capable of analyzing the content of PDFs directly, often without the need for neural models.

At the second stage, the output from pre-processing undergoes more refined analysis using various approaches that can be broadly categorized as rule-based, statistical machine learning, or neural network-based methods. Recent studies have increasingly employed prompt engineering, likely due to its versatility; leveraging large language models (LLMs) such as GPT-3.5 and Mistral. For processing figures and tables, custom vision models have been developed, as seen in the works of Smock et al. (2022) and Khandokar and Deshpande (2024), where Convolutional Neural Networks (CNNs) are used to extract structured data from visual elements in PDFs. Additionally, other neural and statistical models such as Long Short-Term Memory (LSTM) networks, Support Vector Machines (SVMs), Conditional Random Fields (CRFs), and their variants have also been utilized at this stage, as demonstrated in studies like Siciliani et al. (2024), Palm et al. (2019), or Adamson et al. (2023).

### 5.3 Representation and storage of the extracted information

Regarding data storage, the reviewed studies explored a range of methods for storing information extracted from PDF documents. One of the most commonly used formats is JavaScript Object Notation (JSON), which enables the representation of complex, hierarchical data structures in a human-readable and machine-processable textual format. JSON is widely supported across most programming languages, offering efficient parsing and integration capabilities that make it a popular choice for storing structured information (Yehia et al., 2019; Zhu and Cole, 2022; Khandokar and Deshpande, 2024). Closely following JSON in popularity is Extensible Markup Language (XML), which provides similar advantages in terms of flexibility, readability, and cross-platform compatibility (Ahmed and Afzal, 2020; Li et al., 2019; Smock et al., 2022). Both JSON and XML serve as foundational formats for structuring extracted information, but they are often used in conjunction with more advanced storage solutions. These include relational databases, graph databases, or NoSQL databases, which support sophisticated query languages such as SQL and SPARQL. Such technologies facilitate efficient data retrieval, enable complex analytical tasks, and support knowledge discovery by linking extracted data in meaningful ways. In terms of data representation, many studies adopt the use of knowledge triplets (subject-predicate-object) structures that form the backbone of knowledge graphs. This representation allows for the semantic linking of entities and relationships, making the stored information not only more interpretable but also more useful for downstream reasoning, integration, and analysis tasks.

### 5.4 Performance evaluation

Named Entity Recognition (NER) emerged as one of the most frequently performed tasks, often serving as a foundational step in the information extraction pipeline. This was closely followed by Relation Extraction (RE), which builds on the output of NER to identify and classify relationships between recognized entities. Together, these tasks are central to transforming unstructured text into structured knowledge representations. To assess the performance of the proposed models, most studies relied on standard evaluation metrics such as accuracy, precision, recall, and F1-score (Nundloll et al., 2022; Khandokar and Deshpande, 2024; Gemelli et al., 2022). These metrics are widely adopted in the NLP community for their effectiveness in quantifying model performance, especially in classification tasks like NER and RE. Precision and recall provide insight into a model's ability to correctly identify relevant entities and relationships while minimizing false positives and false negatives, respectively. The F1-score, as a harmonic mean of precision and recall, offers a balanced view of a model's overall effectiveness. Accuracy, though slightly less informative in imbalanced datasets, was also commonly reported. These evaluation strategies reflect a shared emphasis on rigorous quantitative assessment to validate the reliability and generalizability of the developed systems. Due to differences in objectives, methods, and datasets across the surveyed works, direct comparative benchmarking was not practical.

### 5.5 Identified challenges

Despite notable advancements in information extraction techniques, a range of persistent challenges continues to hinder the development of universally robust and scalable systems. A core issue lies in the inherent complexity and variability of PDF documents. These documents often exhibit inconsistent formatting, irregular layouts, and structural heterogeneity not only across different domains but even within the same document type (Nundloll et al., 2022; Zhu and Cole, 2022; Ahmed and Afzal, 2020; Khandokar and Deshpande, 2024). Examples include the variability in financial tables or the diverse stylistic conventions found in clinical notes. This inconsistency makes it difficult to design extraction systems that generalize well across formats. One of the most frequently encountered technical obstacles is the handling of PDF files. Originally designed for fixed visual presentation rather than structured data representation, PDFs complicate the reliable extraction of text and structural elements such as tables, figures, and section hierarchies. The challenge becomes even more pronounced in the case of scanned or historical documents, where issues such as poor image quality, handwritten content, and non-standard typography further impair automated analysis.

Compounding these difficulties is the prevalence of domain-specific language, specialized terminology, and abbreviations, which often cannot be accurately interpreted by general-purpose NLP models (Becker et al., 2019; Yoo et al., 2022). Understanding such language frequently requires domain expertise, tailored linguistic resources, or custom-trained models. Additionally, the inherent ambiguity and context dependency of natural language demand sophisticated models capable of nuanced interpretation. Another critical barrier is the lack of large-scale, high-quality annotated datasets tailored to specific domains and document types. The creation of these datasets is both resource-intensive and time-consuming, limiting the availability of labeled data necessary for training supervised learning models and thereby restricting the genericity and accuracy of extraction systems. Beyond these technical challenges, there are operational concerns related to scalability and computational efficiency, particularly when processing high volumes of documents. Evaluating the accuracy and completeness of extracted information remains difficult, often requiring laborious manual validation. Furthermore, maintaining and updating domain ontologies and knowledge graphs introduces additional complexity, especially in rapidly evolving fields. Redundancy and inconsistency in the outputs of Open Information Extraction systems also remain unresolved issues that require targeted mitigation strategies. In sum, while recent innovations have significantly advanced the field, the multifaceted nature of unstructured documents and the demands for scalable, accurate, and domain-adaptable solutions continue to drive ongoing research and development.

## 6 The novel conceptual framework for information extraction from PDF documents

**Figure 5:**
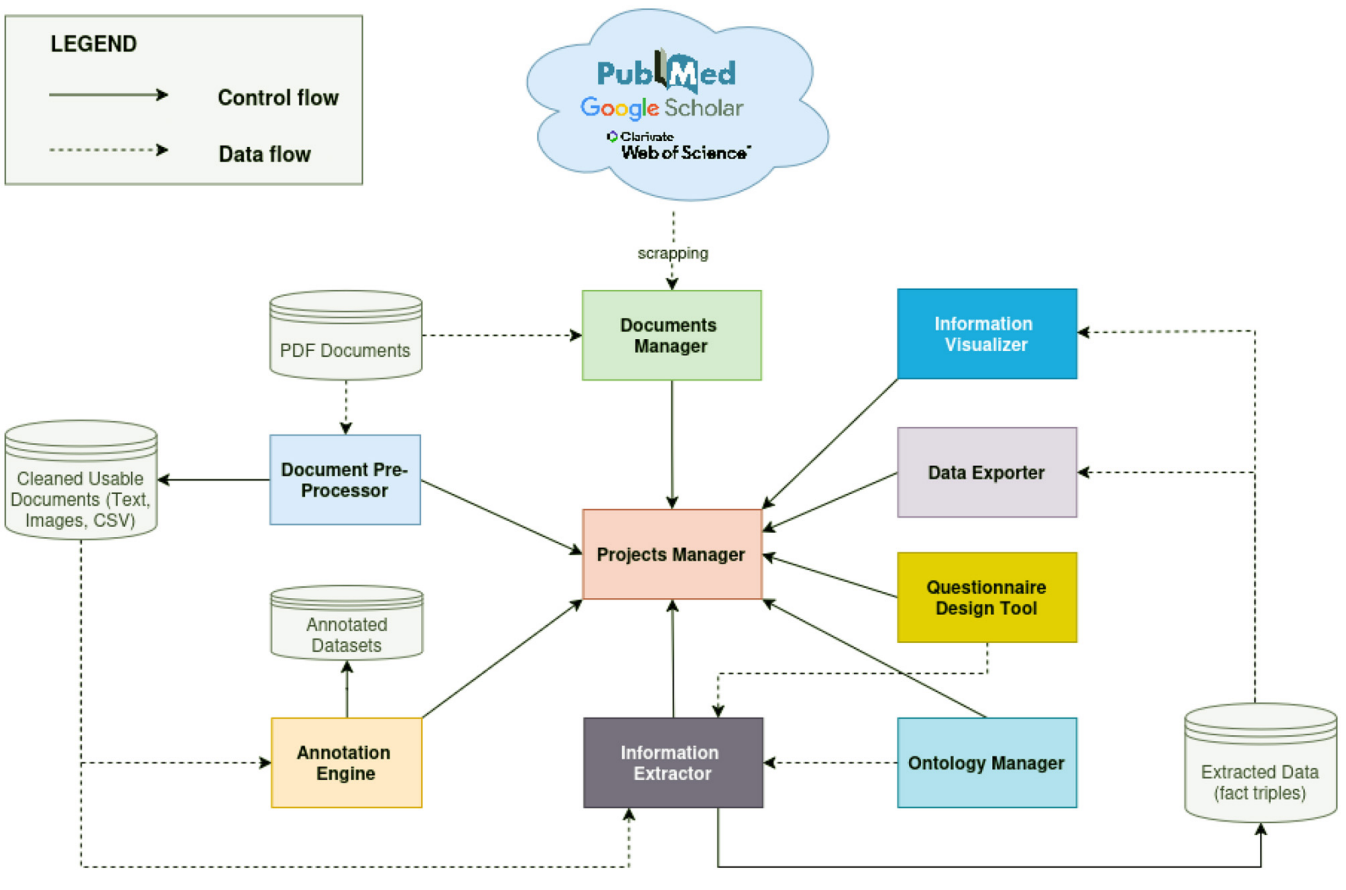
An overview of the proposed data extraction framework, combining common ontologies and language models to improve the flexibility of the information extraction system. *Control flow* illustrates how modules intercommunicate and *data flow* illustrates how data circulates within the system.

IE offers significant potential for enhancing data discovery across various domains. However, existing solutions often exhibit domain-specificity and limited adaptability or requirements of expert knowledge to guide the models in the specific case of LLMs, which leverages prompt engineering and finetuning. To address these challenges, we propose an integrated framework that leverages the strengths of both rule-based and automatic learning-based approaches (see Figure 5). This hybrid approach aims to reduce the reliance on extensive training datasets or expertise. Furthermore, we advocate for the integration of language models and common ontologies (Abhilash and Mahesh, 2023), facilitating cross-domain adaptability and mitigating the need for large training datasets. Our envisioned framework comprises nine modules:

i) A *Projects manager*: This module enables users to create and manage multiple information extraction projects. Each IE exercise is considered an independent project, with its own set of documents and extraction objectives.
ii) A *Documents manager*: This module will enable users to build and manage a document database by either uploading local files or querying online academic libraries such as PubMed, Web of Science, and Google Scholar. Ideally, the module should support web scraping and API integration to retrieve relevant articles based on user-defined keywords and timeframes. Users should be able to seamlessly import documents from both online sources and local storage. Additionally, the system should provide robust tools for organizing, reading, and visualizing extracted information from PDF documents (i.e., outputs from the document pre-processor), facilitating efficient document handling and validation of the extracted content.
iii) A *Document pre-processor*: This module will leverage OCR engines, PDF text extraction libraries (e.g., PyPDF, PyMuPDF), table extraction models, and figure extraction tools to convert PDF documents in the database into more usable components such as plain text, extracted images, and tabular data in CSV format.
iv) An *Ontology manager*: This module will allow users to define and manage the core vocabulary for the Information Extraction (IE) system. This vocabulary will be used to annotate the extracted information in alignment with domain-specific knowledge. Within this module, users will be able to import, visualize, and edit ontology concepts and relationships through an intuitive interface. These ontologies will serve as the backbone of the system and should be associated with specific extraction tasks, enabling more accurate and context-aware information processing.
v) An *Annotation engine*: This module will enhance the accuracy of the IE system by enabling users to create custom annotation datasets based on common ontology concepts and relationships. These datasets will then be used to fine-tune the system's language model, making it more accurate for the specific domain.
vi) A *Questionnaire design tool*: This module will provide a user-friendly interface for defining natural language questions in order to clearly define the scope of the extraction task.
vii) An *Information extractor module*: This module will combine rule-based and language model–driven approaches, harnessing state-of-the-art LLMs such as Google Gemini (Team et al., 2023) or LLaMA (Touvron et al., 2023) to extract specific information. Its primary function is to extract, filter, and align fact triples that fall within the scope defined by users through the questions designed in the Questionnaire Design module.
viii) A *Knowledge visualizer*: This module will facilitate the exploration of structured data through a knowledge graph representation and may include automatic reasoning algorithms to enable knowledge querying and discovery, together with tabular representation of data.
ix) A *Data export module*: This module will allow users to download the extraction results in CSV format to facilitate the inter-operability of this proposed system with third party systems better suited for processing or analyzing the extracted data.

In summary, our framework integrates document annotation, language models, and ontologies to enhance domain adaptability and customization. This combined approach presents significant potential for improving the performance and flexibility of information extraction systems. Recent progress in natural language processing, particularly the integration of large language models with standardized ontologies, offers promising pathways to boost both the accuracy and efficiency of IE systems across diverse application domains. However, the issues with LLM context length could be addressed by incorporating approaches such as PDFTriage proposed by Saad-Falcon et al. (2023), or utilizing PDF-WuKong proposed by Xie et al. (2024) for more accurate PDF understanding and LLM question answering.

The holistic, all-in-one perspective of our proposed framework stems from the growing need to construct structured databases from large collections of unstructured PDF documents. While LLMs represent the most advanced tools for natural language understanding developed to date, their limited controllability by non-expert users poses a challenge to broader adoption. Although prompt engineering provides a means to guide their behavior, it requires expertise that many domain users may lack. To address this, we propose to focus on knowledge triple extraction, enabling the development of a standardized extraction pipeline and uniform formatting of results. Common ontologies primarily serve to uniformize the formatting of results by defining standardized relationships between concepts within a specific domain. Subsequently, these defined concepts and relationships can be leveraged to classify entities and relationships extracted from textual data, thereby yielding more precise and domain-relevant information. By combining LLMs with domain-specific ontologies, our framework aims to bridge this usability and domain adaptability gap. This integration aims to enable a shared understanding of data formats between the information extractor and the language model, thereby facilitating more controlled and semantically consistent extraction outcomes. This combination has been explored in recent works such as Rula and D'Souza (2023), which used the GPT-4 model to extract procedures from unstructured PDF documents through an incremental question-answering approach. They explored zero-shot and in-context learning scenarios, customizing GPT-4 with an ontology of procedures/steps and few-shot learning examples. Their results highlight the potential of LLMs for procedural text mining, with in-context learning significantly improving performance, especially in ontology applications.

## 7 Conclusion

Information Extraction has garnered significant attention due to the prevalence of unstructured data in natural language text. While impressive solutions have been developed across various domains, several challenges persist in achieving highly reliable IE systems, particularly when extracting information from complex PDF documents. These challenges include the intricate and time-intensive nature of building rule-based systems, the scarcity of well-annotated datasets for automatic learning approaches, and the complexity of handling text ambiguity, and semantic especially in very long texts. To address these challenges and promote adaptability across domains, we have introduced a conceptual hybrid framework that integrates the advantages of these two main categories, with a focus on leveraging common ontologies. Our future endeavors will involve implementing and evaluating the proposed framework.

## Data Availability

The original contributions presented in the study are included in the article/supplementary material, further inquiries can be directed to the corresponding author.
