# Supplementary material for: A review on knowledge and information extraction from PDF documents and storage approaches
Source: Front Artif Intell. 2025 Sep 5;8:1466092. doi: 10.3389/frai.2025.1466092 (PMC12447192; doi:10.3389/frai.2025.1466092)
Supplement: Supplementary file 1 [file Table_1.pdf]

Supplementary Material

1 SUMMARY OF ARTICLES

Table S1: Summary of included studies, spanning from 2017 to 2025 and illustrating the landscape and evolution of IE from PDF documents, highlighting the methods, method class, and the storage approaches explored in each study.

Author	Domain	Objectives	Method & Class	Representation & Storage
Siciliani et al. (2024)	Public Administration	Extract structured facts from announcements of the Public Administration	• *Method:* Linear Regression + SVM + BERT<br>• *Class:* (Statistical ML + Neural Network)	Fact Triplets
Dong et al. (2021)	Welding industry	Build a welding information database from unstructured welding documents	• *Method:* Combination of Open Source Python libraries (lxml, pymupdf, pdfminer, pydf2, opencv, tesseract, beautifulsoup) + SWEET ontology 2014<br>• *Class:* Statistical ML + Neural Network	JSON + MongoDB
Scannapieco and Tomazzoli (2024)	Document Management	Information extraction from business documents	Verb Based Semantic Role Labeling	JSON
Nundloll et al. (2022)	Ecology & Conservation Science	Extracting valuable floristic information from a historical Botany journal, and organize it in a unified a query-able database	• *Method:* OCR + NER Model<br>• *Class:* Neural Network	JSON + Mongo DB

Continued on next page

Table S1 – continued from previous page

Author	Domain	Objectives	Method & Class	Representation & Storage
Becker et al. (2019)	Health	Identify specific guideline-based patient information and to annotate it with Unified Medical Language System (UMLS) concepts for manual evaluation by a physician from unstructured sources.	• *Method:* OCR + Supervised learning Model + Rule base system for domain concept alignment<br>• *Class:* Rule-based + Neural Network	UMLS Ontology
Yehia et al. (2019)	Health	Develop an ontology-based clinical information extraction system (OB-CIE), for extracting clinical concepts from physician's free-text notes and converts the unstructured clinical notes to structured information to be accessed in electronic health records	• *Method:* OCR + OpenNLP + Ontology-based NER<br>• *Class:* Rule-based + Neural Network	JSON
Abulaish et al. (2019)	Health	Identify and extract disease symptoms and their associations from biomedical text documents retrieved from the PubMed database.	• *Method:* POS Tagging + MetaMap<br>• *Class:* Rule-based	Semantic Triples
Tao et al. (2024)	Geology	Building an efficient workflow for automatically extracting mineral exploration information from unstructured geological text data using a deep learning method.	• *Method:* PyMuPDF + transformer model (BERT) layer + , Bi-LSTM + CNN + Multi-Head Attention fusion layer + Boundary Prediction + CRF layer<br>• *Class:* Neural Network	Text + Word cloud
Zhu and Cole (2022)	Chemistry	Improve ChemDataExtractor a tools for Chemistry domain NER with ability to process PDF documents, but extracting and labeling PDF text and it structure.	• *Method:* PDFMiner + Custom extraction pipelines<br>• *Class:* Rule-Based	JSON & Plain Text

Continued on next page

Table S1 – continued from previous page

Author	Domain	Objectives	Method & Class	Representation & Storage
Yoo et al. (2022)	Health	Demonstrate the applicability of the OMOP CDM oncology extension module for thyroid cancer diagnosis and cancer stage information by processing free-text medical reports	• *Method:* Rule-based NER + Ontology<br>• *Class:* Rule-based	Labeled concepts in Plain Text, loaded in an OMOP CDM relational database
Afzal et al. (2017)	Health	Data extraction from unstructured clinical records	• *Method:* Rule-based NER<br>• *Class:* Rule-based	Text + Relational Database
Li et al. (2019)	Health	Figure and caption extraction from biomedical documents	• *Method:* Xpdf 2014<br>• *Class:* Rule-based	HTML, Text-stripped PDF pages for subsequent analysis
Ni et al. (2021)	Health	Detect substance use information from unstructured clinical health records	• *Method:* NLP and machine learning–based substance information screener (SIS) + Logic-based rule matcher + Ontologies (UMLS, SnoMED, RXNorm)<br>• *Class:* Statistical ML + Rule-Based	Text
Ahmed and Afzal (2020)	Scientific publishing	Map the Research Articles Sections to IMRAD (Introduction, Methods, Results, And Discussion)	• *Method:* PDFX + XPath/XQuery + SQL 2013<br>• *Class:* Rule-based	XML + PostgreSQL
Zhao et al. (2020)	Information Extraction	Propose an information extraction model and constructs a domain knowledge graph for technical documents	• *Method:* Word2Vec's CBOW Model + TextCNN Model + Neo4J<br>• *Class:* Neural Network + Rule-based	Knowledge triplets + Neo4j Graph Database
Khandokar and Deshpande (2024)	Economy	Harness computer vision-Based framework to extract data from heterogeneous financial tables	• *Method:* TR-OCR + FR-CNN + Transformer<br>• *Class:* Neural Network	JSON

Continued on next page

Table S1 – continued from previous page

Author	Domain	Objectives	Method & Class	Representation & Storage
Smock et al. (2022)	Information extraction	Comprehensive table extraction from unstructured documents	• *Method:* OCR + Needleman-Wunsch algorithm 2008 + Detection Transformer Model + R-CNN + ResNet-18<br>• *Class:* Neural Network + Rule-based	XML, HTML
Yuan et al. (2020)	Information extraction	Table information extraction from PDF documents	• *Method:* Tesseract OCR + OpenCV region detection<br>• *Class:* Neural Network + Rule-based	Image
Palm et al. (2019)	Information extraction	Information extraction from PDF documents	• *Method:* OCR + CNN + LSTM<br>• *Class:* Neural Network	Text
Yang et al. (2019)	Information extraction	Improve information extraction from PDF document of scientific literature.	• *Method:* PyMuPDF + Naïve Bias + ChemicalTagger 2011<br>• *Class:* Rule-based	XML
Gemelli et al. (2022)	Information extraction	Table extraction from PDF documents	• *Method:* OCR + PyMuDPF + sciBERT + Graph Neural Network<br>• *Class:* Neural Networks	Graph data structure
Parrolivelli and Stanchev (2023)	Information extraction	Relationship extraction from unstructured documents	• *Method:* CNN + AllenNLP + LUKE-based transformer<br>• *Class:* Neural Network	Text, Knowledge triplets, Knowledge Graph
Crema et al. (2024)	Health	Develop an automated end-to-end pipeline that extracts relevant information from clinical documents and stores it in a centralized REDCap database	• *Method:* finetuned BERT model<br>• *Class:* Neural Network	JSON + REDCap database

Continued on next page

Table S1 – continued from previous page

Author	Domain	Objectives	Method & Class	Representation & Storage
Cesista et al. (2024)	Information extraction	Business documents information extraction	• *Method:* OCR + LLM Prompt Engineering (Hermes 2 Pro - Mistral 7B, GPT-3.5)<br>• *Class:* Neural Network	JSON
Salamanca et al. (2024)	Archives management	Develop a supervised pipeline for extracting and structuring of content of archival records	• *Method:* Tesseract OCR + CRF<br>• *Class:* Neural Network + Statistical ML	XML
Adamson et al. (2023)	Health	Extraction of real-world data variables from electronic health records	• *Method:* OCR + LSTM<br>• *Class:* Neural Network	Labeled data (JSON / XML)
Jaberi-Douraki et al. (2021)	Animal health	Develop and evaluate a data-mining method for automatically extracting rapid assay data from electronic documents	• *Method:* PyPDF2 + BeautifulSoup<br>• *Class:* Rule-based	Spreadsheets
Papadopoulos et al. (2020)	Information extraction	Propose a novel methodology for Open Information Extraction (OIE) from scientific documents.	• *Method:* Coreference resolution with AllenNLP + Text summarization with sciBERT + triplet extraction (ClausIE + AllenNLP)<br>• *Class:* Statistical ML + Neural Network	JSON + Knowledge triplets
Cho et al. (2023)	Economy	Propose a multi-modal approach-based intelligent document processing framework that combines a pre-trained deep learning model with traditional robotic process automation (RPA) used in banks to automate business processes from real-world financial document images	• *Method:* OCR + Transformer-based model + XLM-RoBERTa + InfoXLM<br>• *Class:* Neural Network	XML

Continued on next page

Table S1 – continued from previous page

Author	Domain	Objectives	Method & Class	Representation & Storage
Tian et al. (2024)	Adminis-tration	Intelligent extraction of key information from Customs Unstructured Accompanying Documents, in regard to security risk identification.	• *Method:* OCR + CNN<br>• *Class:* Neural Network	Text, XML, Knowledge triplets
Saad-Falcon et al. (2023)	Information Extraction	Enables LLMs to retrieve the context based on either structure or content of PDF documents to improve question answering (QA) accuracy over them.	• *Method:* Adobe Extract API + OpenAI function calling API + GPT-3.5 for QA<br>• *Class:* Neural Network	HTML, JSON
Rula and D'Souza (2023)	Information extraction	explore the usage of LLMs in both zero-shot and in-context learning settings to tackle the problem of extracting procedures from unstructured PDF text in an incremental question-answering fashion.	• *Method:* GPT-4 + Prompt Engineering + Ontology<br>• *Class:* Neural Network	Text
Salloum et al. (2018)	Information extraction	identifying and visualizing the topics of a scientific text related to machine learning research and developing a hierarchical and evolutionary connection among these topics.	• *Method:* RapidMiner + Tokenization + Text Clustering + Association Rule Extraction + K-Means Algorithm + Word Cloud<br>• *Class:* Rule based	Word Cloud, Network Graph (Concept link diagram, Similarity diagram)
Xie et al. (2024)	Information extraction	Enhance question-answering capabilities of LLMs on large PDF documents	• *Method:* Sparse Sampler + Multi-Modal-LLM<br>• *Class:* Neural Network	Text

REFERENCES

Abulaish, M., Parwez, M. A., et al. (2019). Disease: A biomedical text analytics system for disease symptom extraction and characterization. *Journal of Biomedical Informatics* 100, 103324

Adamson, B., Waskom, M., Blarre, A., Kelly, J., Krismer, K., Nemeth, S., et al. (2023). EnglishApproach to machine learning for extraction of real-world data variables from electronic health records. *Frontiers in Pharmacology* 14. doi:10.3389/fphar.2023.1180962. Publisher: Frontiers

Afzal, M., Hussain, M., Khan, W. A., Ali, T., Jamshed, A., and Lee, S. (2017). Smart extraction and analysis system for clinical research. *Telemedicine and e-Health* 23, 404–420

Ahmed, I. and Afzal, M. T. (2020). A systematic approach to map the research articles' sections to imrad. *IEEE Access* 8, 129359–129371

Becker, M., Kasper, S., Böckmann, B., Jöckel, K.-H., and Virchow, I. (2019). Natural language processing of german clinical colorectal cancer notes for guideline-based treatment evaluation. *International journal of medical informatics* 127, 141–146

Cesista, F. L., Aguiar, R., Kim, J., and Acilo, P. (2024). Retrieval augmented structured generation: Business document information extraction as tool use. In *2024 IEEE 7th International Conference on Multimedia Information Processing and Retrieval (MIPR)* (IEEE), 227–230

Cho, S., Moon, J., Bae, J., Kang, J., and Lee, S. (2023). A framework for understanding unstructured financial documents using rpa and multimodal approach. *Electronics* 12, 939

Constantin, A., Pettifer, S., and Voronkov, A. (2013). Pdfx: fully-automated pdf-to-xml conversion of scientific literature. In *Proceedings of the 2013 ACM symposium on Document engineering*. 177–180

Crema, C., Verde, F., Tiraboschi, P., Marra, C., Arighi, A., Fostinelli, S., et al. (2024). Medical information extraction with nlp-powered qabots: A real-world scenario. *IEEE Journal of Biomedical and Health Informatics*

DiGiuseppe, N., Pouchard, L. C., and Noy, N. F. (2014). Sweet ontology coverage for earth system sciences. *Earth Science Informatics* 7, 249–264

Dong, Z., Paul, S., Tassenberg, K., Melton, G., and Dong, H. (2021). Transformation from human-readable documents and archives in arc welding domain to machine-interpretable data. *Computers in Industry* 128, 103439

Gemelli, A., Vivoli, E., and Marinai, S. (2022). Graph neural networks and representation embedding for table extraction in pdf documents. In *2022 26th International Conference on Pattern Recognition (ICPR)* (IEEE), 1719–1726

Hawizy, L., Jessop, D. M., Adams, N., and Murray-Rust, P. (2011). Chemicaltagger: A tool for semantic text-mining in chemistry. *Journal of cheminformatics* 3, 1–13

Jaberi-Douraki, M., Taghian Dinani, S., Millagaha Gedara, N. I., Xu, X., Richards, E., Maunsell, F., et al. (2021). Large-scale data mining of rapid residue detection assay data from html and pdf documents: improving data access and visualization for veterinarians. *Frontiers in veterinary science* 8, 674730

Khandokar, I. A. and Deshpande, P. (2024). Computer vision-based framework for data extraction from heterogeneous financial tables: A comprehensive approach to unlocking financial insights. *IEEE Access*

Li, P., Jiang, X., and Shatkay, H. (2019). Figure and caption extraction from biomedical documents. *Bioinformatics* 35, 4381–4388

Likic, V. (2008). The needleman-wunsch algorithm for sequence alignment. *Lecture given at the 7th Melbourne Bioinformatics Course, Bi021 Molecular Science and Biotechnology Institute, University of Melbourne* , 1–46

Ni, Y., Bachtel, A., Nause, K., and Beal, S. (2021). Automated detection of substance use information from electronic health records for a pediatric population. *Journal of the American Medical Informatics Association* 28, 2116–2127

Nundloll, V., Smail, R., Stevens, C., and Blair, G. (2022). Automating the extraction of information from a historical text and building a linked data model for the domain of ecology and conservation science. *Heliyon* 8

Palm, R. B., Laws, F., and Winther, O. (2019). Attend, copy, parse end-to-end information extraction from documents. In *2019 International Conference on Document Analysis and Recognition (ICDAR)* (IEEE), 329–336

Papadopoulos, D., Papadakis, N., and Litke, A. (2020). A methodology for open information extraction and representation from large scientific corpora: the cord-19 data exploration use case. *Applied Sciences* 10, 5630

Parrolivelli, C. and Stanchev, L. (2023). Genealogical relationship extraction from unstructured text using fine-tuned transformer models. In *2023 IEEE 17th International Conference on Semantic Computing (ICSC)* (IEEE), 167–174

Rula, A. and D'Souza, J. (2023). Procedural Text Mining with Large Language Models. In *Proceedings of the 12th Knowledge Capture Conference 2023* (New York, NY, USA: Association for Computing Machinery), K-CAP '23, 9–16. doi:10.1145/3587259.3627572

[Dataset] Saad-Falcon, J., Barrow, J., Siu, A., Nenkova, A., Yoon, D. S., Rossi, R. A., et al. (2023). PDFTriage: Question Answering over Long, Structured Documents. doi:10.48550/arXiv.2309.08872. ArXiv:2309.08872 [cs]

Salamanca, L., Brandenberger, L., Gasser, L., Schlosser, S., Balode, M., Jung, V., et al. (2024). Processing large-scale archival records: The case of the swiss parliamentary records. *Swiss Political Science Review* 30, 140–153

Salloum, S. A., Al-Emran, M., Monem, A. A., and Shaalan, K. (2018). Using text mining techniques for extracting information from research articles. *Intelligent natural language processing: Trends and Applications* , 373–397

Scannapieco, S. and Tomazzoli, C. (2024). Cnosso, a novel method for business document automation based on open information extraction. *Expert Systems with Applications* 245, 123038

Siciliani, L., Ghizzota, E., Basile, P., and Lops, P. (2024). Oie4pa: open information extraction for the public administration. *Journal of Intelligent Information Systems* 62, 273–294

Smock, B., Pesala, R., and Abraham, R. (2022). Pubtables-1m: Towards comprehensive table extraction from unstructured documents. In *Proceedings of the IEEE/CVF Conference on Computer Vision and Pattern Recognition*. 4634–4642

Tao, J., Zhang, N., Chang, J., Chen, L., Zhang, H., Liao, S., et al. (2024). Deep learning-based mineral exploration named entity recognition: A case study of granitic pegmatite-type lithium deposits. *Ore Geology Reviews* , 106367

Tian, F., Wang, H., Wan, Z., Liu, R., Liu, R., Lv, D., et al. (2024). Unstructured document information extraction method with multi-faceted domain knowledge graph assistance for m2m customs risk prevention and screening application. *Electronics* 13, 1941

Xie, X., Yan, H., Yin, L., Liu, Y., Ding, J., Liao, M., et al. (2024). Wukong: A large multimodal model for efficient long pdf reading with end-to-end sparse sampling. *arXiv preprint arXiv:2410.05970*

Yang, H., Aguirre, C. A., Maria, F., Christensen, D., Bobadilla, L., Davich, E., et al. (2019). Pipelines for procedural information extraction from scientific literature: towards recipes using machine learning and data science. In *2019 International conference on document analysis and recognition workshops (ICDARW)* (IEEE), vol. 2, 41–46

Yang, X., Juhas, P., Farrow, C. L., and Billinge, S. J. (2014). xpdfsuite: an end-to-end software solution for high throughput pair distribution function transformation, visualization and analysis. *arXiv preprint arXiv:1402.3163*

Yehia, E., Boshnak, H., AbdelGaber, S., Abdo, A., and Elzanfaly, D. S. (2019). Ontology-based clinical information extraction from physician's free-text notes. *Journal of biomedical informatics* 98, 103276

Yoo, S., Yoon, E., Boo, D., Kim, B., Kim, S., Paeng, J. C., et al. (2022). Transforming thyroid cancer diagnosis and staging information from unstructured reports to the observational medical outcome partnership common data model. *Applied Clinical Informatics* 13, 521–531

Yuan, J., Li, H., Wang, M., Liu, R., Li, C., and Wang, B. (2020). An opencv-based framework for table information extraction. In *2020 IEEE International Conference on Knowledge Graph (ICKG)* (IEEE), 621–628

Zhao, H., Pan, Y., and Yang, F. (2020). Research on information extraction of technical documents and construction of domain knowledge graph. *Ieee Access* 8, 168087–168098

Zhu, M. and Cole, J. M. (2022). Pdfdataextractor: A tool for reading scientific text and interpreting metadata from the typeset literature in the portable document format. *Journal of Chemical Information and Modeling* 62, 1633–1643
